# SlAREB1 regulates ethylene biosynthesis and mediates the effect of abscisic acid on postharvest ripening of tomato fruit

**DOI:** 10.3389/fpls.2026.1773076

**Published:** 2026-05-04

**Authors:** Jianghui Huang, Qiong Wu, Yanan He, Dongdong Zhang, Yurong Zhang, Xiaoya Tao

**Affiliations:** 1 School of Food and Strategic Reserves, Collaborative Innovation Center of Henan Grain Crops, Grain Storage and Security Engineering Research Center of Education Ministry, Henan University of Technology, Zhengzhou, China; 2 College of Food Science and Technology, Nanjing Agricultural University, Nanjing, China

**Keywords:** abscisic acid, ethylene biosynthesis, hormonal interactions, SlAREB1, tomato fruit ripening

## Abstract

Ethylene biosynthesis during postharvest ripening of tomato fruit is stimulated by the phytohormone abscisic acid (ABA). This research utilized mature green cherry tomato fruit to elucidate the molecular mechanism of SlAREB1 regulating ethylene biosynthesis and mediating ABA’s effect on postharvest ripening of tomato fruit. Results indicated that ABA treatment expedited alterations in fruit color, firmness, ethylene release rate, and modulated the expression of *SlAREB1* and genes associated with ethylene biosynthesis. Meanwhile, SlAREB1 was proven to participate in the regulatory process of ABA on tomato fruit ripening. The results of yeast one-hybrid and dual luciferase assays demonstrated that SlAREB1 interacted with the promoters of *ACS2*, *ACO1*, *FUL1*, and *MADS1*, and activated their transcriptions, thereby regulating ethylene biosynthesis and subsequently affecting the ripening process of postharvest tomato fruit. The molecular model illustrates that SlAREB1 is a downstream component of ABA signaling that directly regulates ethylene-related genes, thereby mediating ABA’s effect. These findings provide a theoretical basis for further improving the regulatory network of hormone interactions during tomato fruit ripening.

## 1 Introduction

Fruit ripening has attracted considerable attentions due to its unique developmental processes in plant biology and its substantial effects on fruit quality and postharvest longevity, encompassing alterations in various attributes of appearance and flavor, such as color, texture, taste, and scent (Kou et al., 2021; Wang et al., 2014b). Fruit development and ripening are meticulously managed temporally and spatially by a complex interplay of numerous plant hormones, influencing fruit quality, nutrition and flavor (Batista-Silva et al., 2022). Fresh fruit can be categorized into climacteric and non-climacteric types based on whether they exhibit respiration and ethylene production during the ripening process (Wang et al., 2014b; Wu et al., 2018a). Tomato is a crucial crop variety globally and serves as a model plant for studying climacteric fruit development and ripening processes, which is attributed to its economic significance, distinct ripening period, brief life cycle, extensive genomic data, and notable metabolic alterations (Li et al., 2022; Tao et al., 2022a; Vitale et al., 2014).

Ethylene is well-known involved in the regulation of tomato fruit ripening and quality traits (Liu et al., 2015). The 1-amino-cyclopropane-1-carboxylic acid (ACC) synthase (ACS) and ACC oxidase (ACO) are two essential enzymes involved in ethylene biosynthesis (Isaacson et al., 2002; Kou et al., 2018), and ACS is considered to be the rate-limiting enzyme for ethylene biosynthesis during tomato fruit ripening (Chen et al., 2022; Yu et al., 2019). Phytohormone abscisic acid (ABA) is mainly involved in regulating non-climacteric fruit ripening. While increasing studies have shown that ABA can promote the ripening of climacteric fruit including tomatoes, bananas, figs, and mangoes (Duan et al., 2015; Jiang and Macnish, 2000; Qiao et al., 2021; Wu et al., 2022; Zhang et al., 2009). Especially, the peak value of endogenous ABA accumulation occurs earlier than that of ethylene production during tomato fruit ripening, indicating that ABA may act as an upstream regulator of the ethylene metabolic pathway (Zhang et al., 2009). Exogenous ABA accelerated tomato fruit ripening by regulating the expressions of multiple genes associated with ethylene biosynthesis and signal pathway (Mou et al., 2015, 2016). Silencing of ABA receptor genes in tomato fruit using RNAi technology revealed that ABA signaling might be upstream of ethylene signaling that regulates fruit ripening, and that ethylene biosynthesis and signal transduction were inhibited in tomato fruit at the early ripening stage, resulting in delayed fruit ripening (Zou et al., 2022). Prior research indicates that ABA promotes ethylene biosynthesis (Mou et al., 2016; Tao et al., 2022b; Wu et al., 2018a, 2018b). However, the precise interaction mechanism between these two hormones during tomato ripening is still ambiguous.

For the downstream target gene expression in ABA signal transduction pathway, the main activation pathway of them was the combination of basic-domain leucine zipper (bZIP) family transcription factors and the ABA-response elements binding factors (AREB/ABFs) within their promoter regions (ABREs, PyACGTGG/TC) (Al-Abdallat et al., 2017; Chen et al., 2020; Fujita et al., 2013; Mou et al., 2018). The transcription factor proteins SlAREB1 and SlAREB2 isolated and identified in tomato can be activated by external ABA, and SlAREB1 has been demonstrated to primarily participate in the expression of stress-related genes and the regulation of the tomato fruit ripening process (Bastias et al., 2011; Orellana et al., 2010; Yanez et al., 2009). ABA signaling mediated by SlAREB1 may modulate fruit ripening-associated metabolic processes with in tomato fruit by triggering the expressions of genes responsible for the biosynthesis of key metabolites, including organic acids (citric acid, malic acid), sugars (glucose, fructose), and amino acids (glutamate) (Bastias et al., 2011, 2014). In comparison to wild-type tomato fruit, the expression levels of ethylene biosynthesis genes (*SlACS2*, *SlACS4*, *SlACO1*, and *SlACO3*) were markedly elevated in *SlAREB1*-overexpressed fruit, whereas those were significantly decreased in SlAREB1-inhibited lines (Bastias et al., 2014). SlAREB1 was proved to interact with SNAC9 and affect ABA signal conduction, further regulating tomato fruit ripening, and the expression of ethylene biosynthesis genes *SlACS2* and *SlACO1* was down-regulated in *SNAC9-*silenced fruit (Yang et al., 2021). These investigations suggest that SlAREB1-mediated ABA signaling may regulate ABA’s influence on ethylene biosynthesis via inducing the expressions of ethylene biosynthesis genes, hence impacting tomato fruit ripening and ripening-related metabolic processes. The transient overexpression of *SlAREB1* in tomato fruit induced caused the up-expression of *NOR* and some ethylene biosynthesis genes including *SlACS2*, *SlACS4*, and *SlACO1*, indicating that SlAREB1 could facilitate ABA signaling to induce *NOR* transcription, hence enhancing ethylene biosynthesis in tomato fruit (Mou et al., 2018).

To date, studies have shown that multiple transcription factors regulate fruit ripening via affecting ethylene biosynthesis. Transcription factors RIN (Li et al., 2011; Vrebalov et al., 2002), CNR (Manning et al., 2006), TAGL1 (Itkin et al., 2009; Vrebalov et al., 2009), and FUL1/FUL2 (Fujisawa et al., 2014; Wang et al., 2014a) are positive regulators, and SlMADS1 is a negative regulator (Dong et al., 2013), all of which are involved in tomato fruit ripening process by directly regulating key genes associated with ethylene biosynthesis pathway or interacting with other key transcription factors to indirectly regulate ethylene biosynthesis. We analyzed the promoters of these known transcription factor genes and found that their promoter sequences contained one or more AREB/ABFs *cis*-regulatory elements, suggesting that they might be regulated by ABA during the tomato ripening process.

This study aims to clarify the molecular mechanism of SlAREB1-mediated the regulation of ABA on ethylene biosynthesis during tomato fruit ripening through identifying the interaction sites between SlAREB1 and ethylene biosynthesis pathway. The results established a theoretical foundation for further elucidating the regulatory network of hormone interactions during postharvest ripening of tomato fruit.

## 2 Materials and methods

### 2.1 Materials and treatments

Mature green cherry tomato fruit (*Solanum lycopersicum* L.) were collected manually in the greenhouse of Henan Aisijia Agricultural Tourism Development Co., Ltd. at Luohe City of China. Fruit with no pests, no surface damage, and uniform size were chosen and transferred to the laboratory within two hours after picking.

Tomato fruit were treated with 1.0 mM ABA solution (98%, HPLC, Aladdin) or sterile distilled water (CK group) under a vacuum condition of 60 kPa for 3 min, and then they were air-dried at room temperature, and finally they were stored in constant temperature incubators for 16 d at 20 °C with 90% relative humidity in the dark as previously outlined (Tao et al., 2020). Samples were taken at 1, 4, 7, 10, 13, and 16 d after treatment, and a total of 24 tomato fruit per treatment group were randomly selected and equally divided into thirds at each sampling time point, frozen in liquid nitrogen and kept at -80 °C for subsequent investigation.

For investigating the short-term effects of ABA on postharvest tomato fruit ripening, two pulp discs were taken from the equatorial regions of each sterile tomato fruit using a 9 mm punch. The discs were then immersed for in either 1 mM ABA or sterile distilled water (CK group) 300 s and kept in the incubator under the above conditions after wiping off the surface liquid with sterile filter paper. Samples were collected at 0, 1, 2, 4, 8, 12, 24, and 48 h after treatment, and kept at -80 °C for gene expression analysis.

To clarify the expression levels of genes in tomato fruit at various growth and developmental periods, tomato fruit at immature green 1 (IMG1), immature green 2 (IMG2), mature green (MG), breaker (Br), turning (T), and red ripe (RR) stages were picked and sampled according to the method described above.

### 2.2 Analysis of fruit coloration, texture, ethylene production and ABA content

Tomato fruit color and firmness were analyzed as per the method described previously (Tao et al., 2020). The results were expressed with a* values, which represent a color component with -a* indicates green and +a* indicates red, and the mean values of the tested highest forces, respectively.

Ethylene production assay and the pre-treatment for ABA determination were conducted based on the previous method (Wu et al., 2018a, 2018b), and the ABA content was determined with high performance liquid chromatography-mass spectrometry (HPLC-MS) and the measurement parameter were set as described previously (Wu et al., 2018a). The fresh weight was used to report the findings as g kg^−1^.

### 2.3 RNA extraction and qRT-PCR

Based on the previous transcriptome data from our laboratory, differentially expressed genes related to ethylene synthesis were selected, including the ethylene synthesis pathway genes *ACS2*, *ACS4*, and *ACO1*; the positive regulator genes *FUL1*, *TAGL1*, *MADS-RIN*, and *CNR*; and the negative regulator gene *MADS1*. Total RNA extraction and qRT-PCR analysis were conducted as per Wu et al. (2025). The relative expressions of target genes were quantified using the 2^-ΔΔCT^ method. The sequences of all the primers utilized in this work were shown in **Supplementary Table 1**.

### 2.4 Yeast one-hybrid assay

Y1H assay was performed as per Wu et al. (2025) using Yeastmaker™ Yeast Transformation System 2 (630439, Takara Biomedical Technology Co., Ltd., Beijing) and Matchmaker™ Insert Check PCR Mix 1 (630496, Takara Biomedical Technology Co., Ltd., Beijing). The promoter sequences of the selected genes were obtained by downloading from the Sol Genomics Network (https://solgenomics.net). The specific location of ABREs was identified using PlantCARE (https://bioinformatics.psb.ugent.be/webtools/plantcare/html/) to determine the target gene fragments. The CDS sequence of *SlAREB1* was cloned into the pGADT7 vector to generate pGADT7-SlAREB1 and used as the prey protein, and fragments of *ACS2*, *ACS4-1*, *ACS4-2*, *ACO1*, *MADS1*, *TAGL1*, *MADS-RIN*, *CNR*, and *FUL1* were individually cloned into the pAbAi vector to generate corresponding bait genes. The pAbAi-bait plasmid was linearized with *BbS Ⅰ* or *BstB I* and transferred into Y1HGold yeast strain. The minimum inhibitory concentration of Aureobasidin A (AbA, Takara Biomedical Technology Co., Ltd., Beijing) for each pAbAi-bait strain was determined on SD/-Ura medium. Briefly, a 100 μL aliquot of the yeast culture was spread onto SD/-Ura selection medium containing a gradient of AbA concentrations (0, 100, 200, 300, and 400 µg L^-1^). The plates were incubated upside down at 30 °C for 3-5 d, and the minimum inhibitory concentration of AbA was determined based on yeast growth conditions. The pGADT7-*SlAREB1* plasmid was then transferred into the Y1HGold strain containing pAbAi-bait, and the DNA-protein interaction was assessed by growth on SD/-Leu medium that contain AbA.

### 2.5 Dual luciferase experiment

The pGreenII;-0800LUC (LUC) vector was used to generate the corresponding reporter vectors pGreenII;-0800LUC-*ACS2 pro* (LUC-*ACS2 pro*), pGreenII;-0800LUC-*ACS4-1 pro* (LUC-*ACS4-1 pro*), pGreenII;-0800LUC-*ACO1 pro* (LUC-*ACO1 pro*), pGreenII;-0800LUC-*FUL1 pro* (LUC-*FUL1 pro*), and pGreenII;-0800LUC-*MADS1 pro* (LUC-*MADS1 pro*). The CDS sequence of *SlAREB1* was cloned into the pGreenII;-62SK (SK) vector to generate the effector vector pGreenII;-62SK-SlAREB1 (SK-SlAREB1). The dual luciferase experiment was conducted as per Wu et al. (2025). Briefly, REN, constitutively driven by the CaMV 35S promoter, was used as an internal control to normalize variations in transformation efficiency and cell viability among different samples. Following co-infiltration into tobacco leaves, the activities of firefly LUC and REN were measured using a dual luciferase reporter assay kit, and the LUC/REN ratio was subsequently calculated.

### 2.6 Statistical analysis

The results were presented as mean ± standard deviation based on three replicates. The significance analysis between the two groups was conducted using IBM SPSS Statistics 22.0 and Student’s *t*-test (*p* < 0.05). Origin2018 was used for plotting figures.

## 3 Results

### 3.1 Effect of exogenous ABA on postharvest tomato fruit ripening

**Figure 1:**
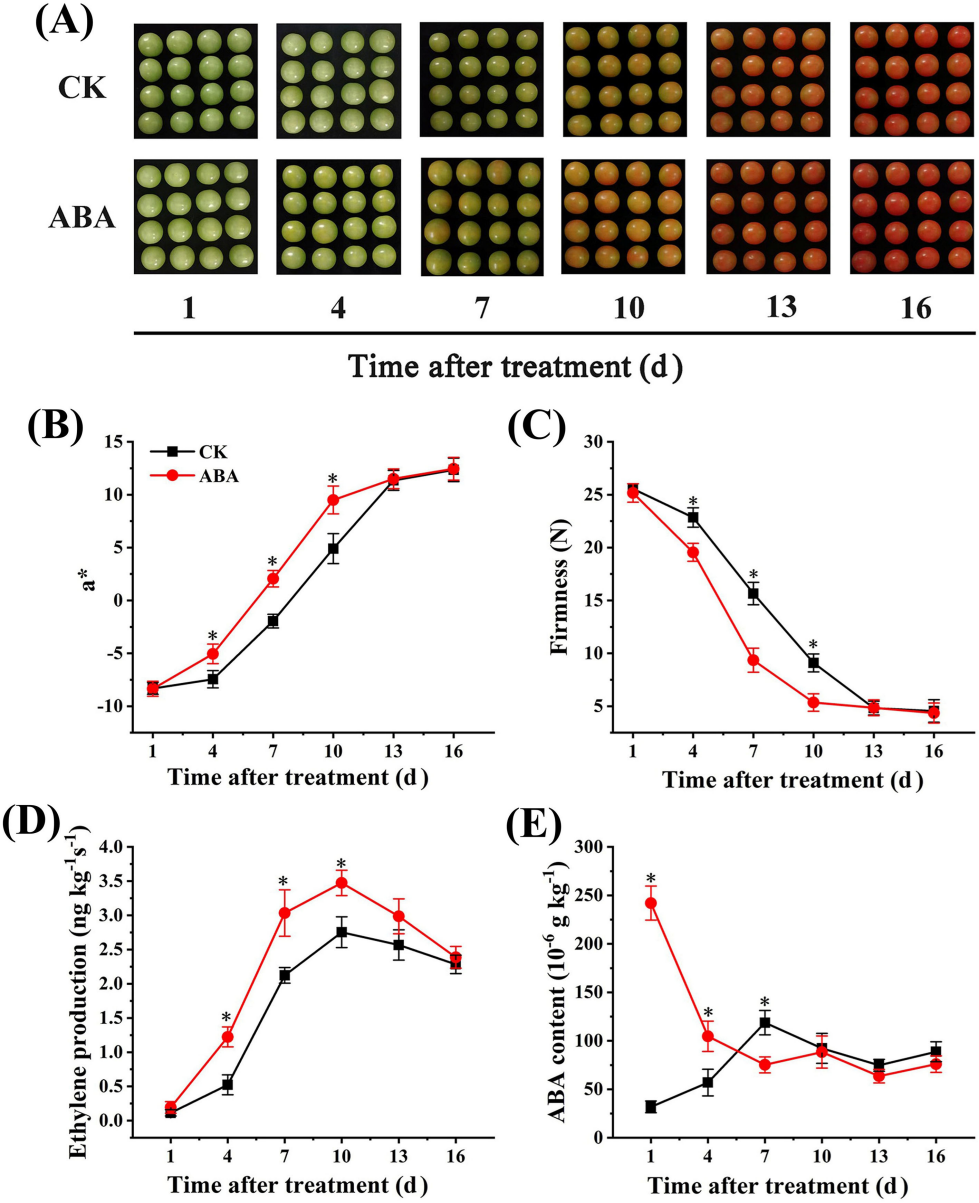
Exogenous ABA affects color phenotypes **(A)**, a* values **(B)**, firmness **(C)**, ethylene production **(D)**, and ABA content **(E)** of tomato fruit during postharvest ripening. The standard error (SE) of three biological replicates was indicated by error bars and asterisks (*) were used to indicate that the values in ABA group were significantly different (*p* < 0.05) from those in CK group.

As shown in **Figures 1A, B**, compared with the fruit in CK group, exogenous ABA treatment accelerated the color change of tomato fruit. The a* value in ABA treated group was significantly higher than that in CK group during 4-10 d, which was 0.68-, 1.1-, and 1.94-fold higher than that in CK group, respectively. No substantial difference was noted at 16 d between the two groups.

The firmness of tomato fruit decreased as fruit ripening (**Figure 1C**). Briefly, the firmness of tomato fruit in ABA treated group was significantly lower than that in CK group during 4-10 d. The firmness of ABA treated fruit was 0.6- and 0.59-fold of that in CK group at 7 d and 10 d, respectively, and no significant difference was observed at 13 d and 16 d between them, suggesting that ABA treatment accelerated tomato fruit softening.

The changes of ethylene release rates in the ABA group and the CK group were similar (**Figure 1D**), both of them reached the peak value at 10 d. The ethylene production in ABA group was significantly higher than that in CK group with peak values of 3.47 ng kg^-1^ s^-1^ and 2.75 ng kg^-1^ s^-1^, respectively, indicating that exogenous ABA promotes the ethylene production of tomato fruit during the ripening process.

As shown in **Figure 1E**, the ABA content in the CK group increased initially and then decreased, and reached the peak value of 118.69 × 10^−6^ g kg^−1^ at 7 d. In ABA treated fruit, it reached the peak value of 242.03 × 10^−6^ g kg^−1^ at 1 d, and decreased afterwards, then reached a small peak again at 10 d.

### 3.2 Expression of *SlAREB1* in response to exogenous ABA and during tomato fruit development and ripening

**Figure 2:**
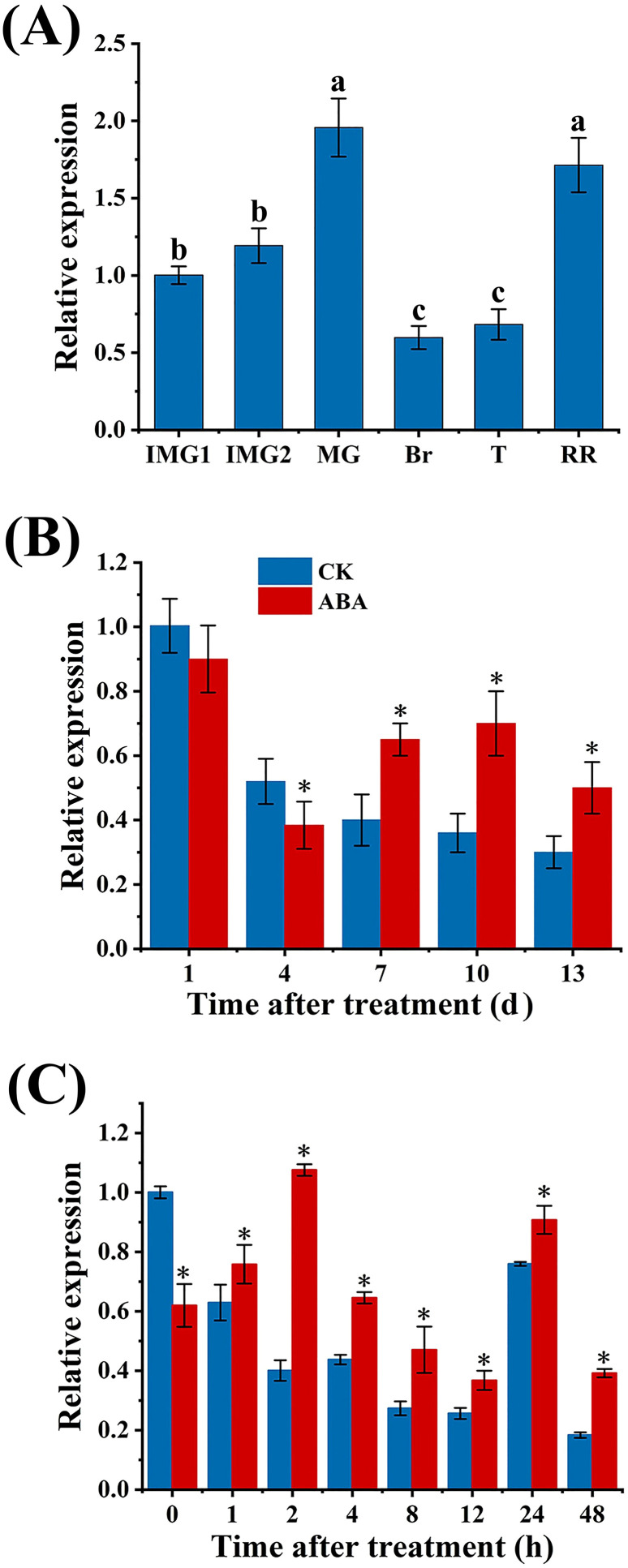
The expression of *SlAREB1* **(A)** during tomato fruit development and ripening and long-term **(B)** and short-term **(C)** effects of exogenous ABA treatment on *SlAREB1*. The standard error (SE) of three biological replicates was indicated by error bars and asterisks (*) were used to indicate that the values in ABA group were significantly different (*p* < 0.05) from those in CK group. Lowercase letters represent the differences from multiple comparisons of *SlAREB1* expression levels at different developmental and ripening stages (*p* < 0.05).

As shown in **Figure 2A**, the expression level of *SlAREB1* progressively grew throughout tomato fruit development, peaked at the mature green stage, subsequently decreased, and then rebounded at the red ripe stage.

The expression level of *SlAREB1* was markedly elevated in ABA group compared with the CK group during 7-13 d (**Figure 2B**). Furthermore, the gene responded rapidly to exogenous ABA treatment, with its expression was higher than that in CK group within 2 h after ABA treatment (**Figure 2C**), indicating that *SlAREB1* may affect the ripening of tomato fruit and engage in the ABA-mediated regulation mechanism of ripening.

### 3.3 Gene promoter analysis

The promoter sequence was determined by analyzing the 2000 bp upstream sequence of the relevant gene encoding regions and their corresponding cDNA sequence. The results of PlantCARE analysis revealed that the AREB elements exist in all the selected tomato fruit ripening and the ethylene biosynthesis related genes promoters (**Table 1**), suggesting that ABA may regulate the expression of these genes.

**Table 1:** Review of *cis*-acting elements found in promoters of genes involved in ethylene biosynthesis.

Gene ID	Gene annotation	Gene length/bp	Site name	Position	Strand	Sequence
Solyc01g095080	*ACS2*	1233	ABRE	1140	+	ACGTG
Solyc05g050010	*ACS4-1*	687	ABRE	150	+	ACGTG
			ABRE	532	+	CACGT
Solyc05g050010	*ACS4-2*	828	ABRE	673	+	ACGTG
Solyc07g049530	*ACO1*	1017	ABRE	691	+	TACGGTC
			ABRE	794	+	CACGT
Solyc03g114840	*MADS1*	755	ABRE	54	+	CGCACGTTTC
			ABRE	266	+	ACGTG
			ABRE	600	+	ACGTG
Solyc07g055920	*TAGL1*	560	ABRE	405	+	ACGTG
Solyc06g069430	*FUL1*	697	ABRE	542	+	ACGTG
Solyc05g012020	*MADS-RIN*	627	ABRE	472	+	ACGTG
Solyc02g077920	*CNR*	374	ABRE	219	+	ACGTG

### 3.4 Effect of exogenous ABA on the expression of key genes associated with ethylene biosynthesis

**Figure 3:**
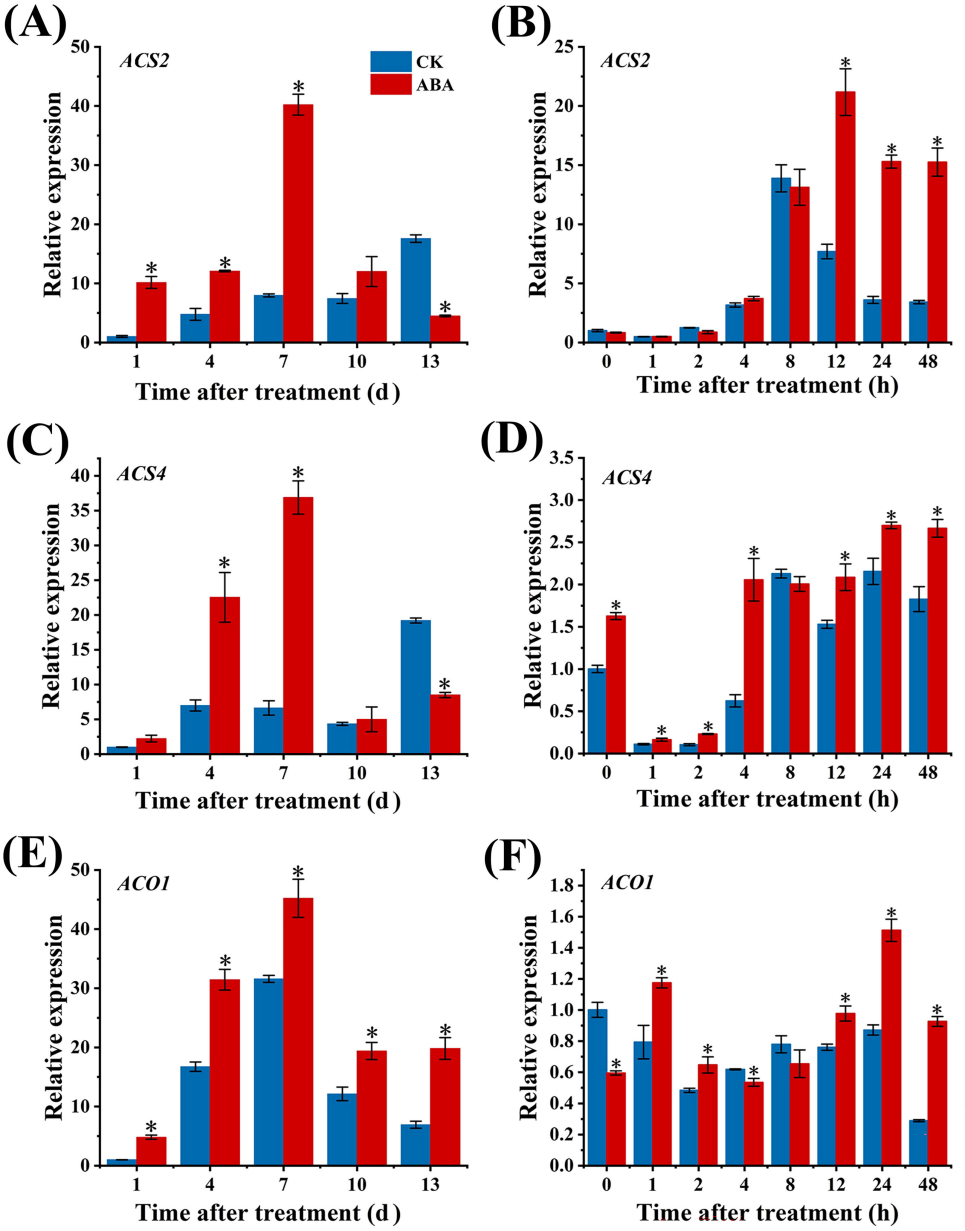
Expression of ethylene biosynthesis-related genes ACS2 **(A)**, ACS4 **(B)**, and ACO1 **(C)** in tomato fruit in response to exogenous ABA treatment. Tomato fruits at the mature green stage were treated with ABA or distilled water (CK). Pericarp samples were collected at 1, 4, 7, 10, and 13 days after treatment. The standard error (SE) of three biological replicates is indicated by error bars. Asterisks (*) indicate significant differences between the ABA-treated group and the CK group at the same time point (p<0.05). Lowercase letters represent significant differences in gene expression levels across different time points within the same treatment group (p<0.05).

As shown in **Figure 3A**, the expression level of *ACS2* in CK group gradually increased during the storage period. In contrast, exogenous ABA treatment induced a biphasic response: *ACS2* expression initially rose, then declined, culminating at 7 d with a magnitude 5-fold higher than that in CK group. The transcription abundances of *ACS2* in both two groups increased first and then decreased afterwards in response to the short-term effect (**Figure 3B**), reaching the peak at 8 h in CK group and 12 h in ABA group. Furthermore, the expression of *ACS2* exhibited a significantly increased level in the ABA group than in the CK group at 12 h after ABA treatment.

Similar to *ACS2*, ABA also increased the expression of *ACS4*, which peaked at 7 d and was 1.4-fold higher than that of the CK group (**Figure 3C**). Additionally, *ACS4* exhibited a rapid response to ABA, maintaining significantly higher expression than that in CK group throughout the 48 h period (except at 8 h) (**Figure 3D**).

As shown in **Figure 3E**, the expression level of *ACO1* in ABA group exceeded that of the CK group, peaking at 7 d. The expression of *ACO1* exhibited a quick response to ABA, with its expression level significantly surpassing that of the CK group at 1 h after ABA treatment (**Figure 3F**).

The results shown above indicate that treatment with exogenous ABA promotes the expression levels of genes associated with the ethylene biosynthesis pathway, including *ACS2*, *ACS4*, and *ACO1*.

### 3.5 Effect of exogenous ABA on the expression of genes related to ripening-related transcription factors in tomato

**Figure 4:**
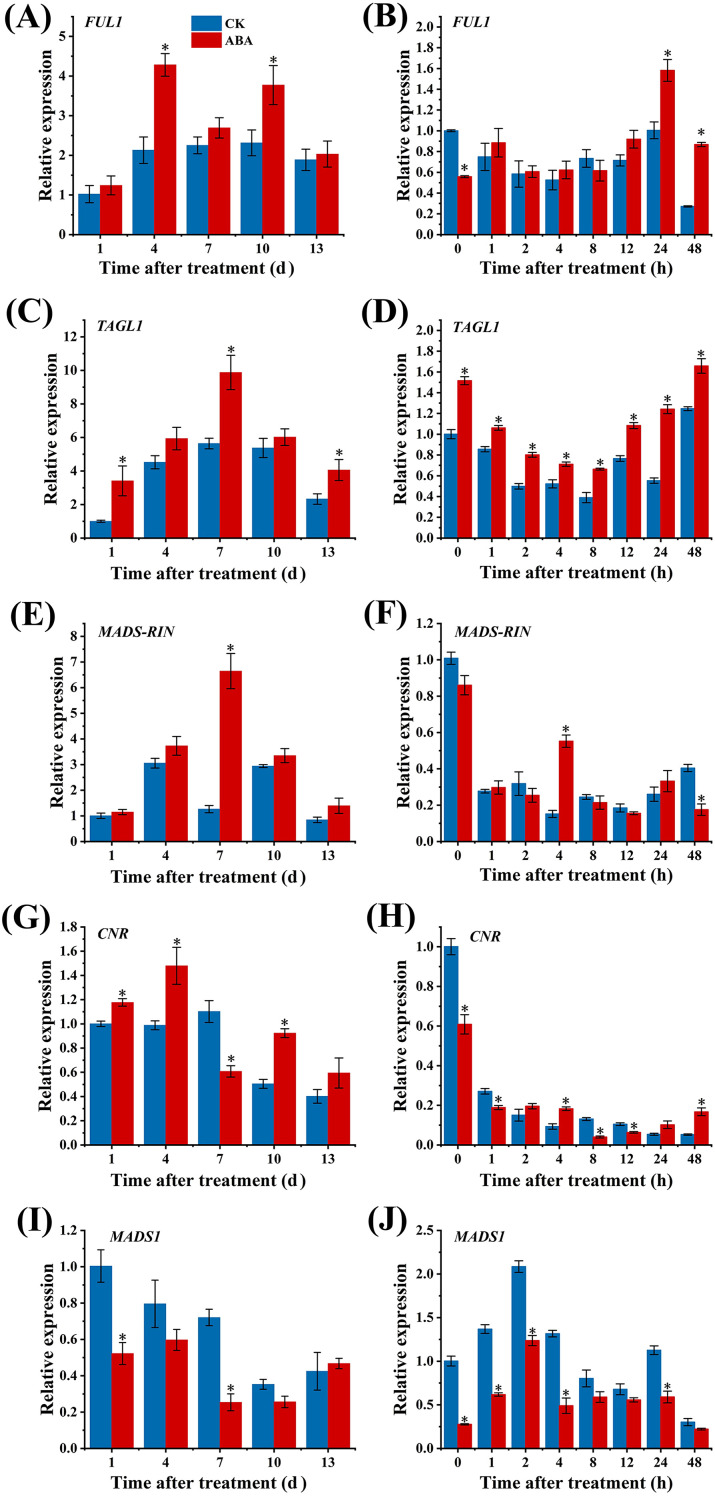
Expression of fruit ripening-related transcription factor genes FUL1 **(A, B)**, TAGL1 **(C, D)**, MADS-RIN **(E, F)**, CNR **(G, H)**, and MADS1 **(I, J)** in tomato fruit in response to exogenous ABA treatment. Tomato fruits at the mature green stage were treated with ABA or distilled water (CK). Pericarp samples were collected at the indicated time points after treatment: 0, 1, 4, 7, 10, 13 days (panels A, C, E, G, I) and 0, 1, 4, 8, 12, 24, 48 days (panels B, D, F, H, J). Relative expression levels were determined by qRT-PCR and normalized to an internal reference gene. The standard error (SE) of three biological replicates is indicated by error bars (if present). Asterisks (*) indicate significant differences between the ABA-treated group and the CK group at the same time point (p<0.05). Lowercase letters represent significant differences in gene expression levels across different time points within the same treatment group (p<0.05).

For the expression of *FUL1*, it remained relatively stable from 4 d to 10 d (**Figure 4A**) in CK group, and its expression level was consistently higher in ABA treated group, reaching a peak value at 4 d with about two folds of that in CK group. The short-term effect results indicated that the *FUL1* gene responded rapidly to ABA, with expression levels constantly higher than that in CK group since 1 h after ABA treatment (**Figure 4B**). Significant differences between the two groups were observed especially at 24 h and 48 h after treatments.

The expression levels of *TAGL1* in two groups exhibited a trend of initially increasing and then decreasing, peaking at 7 d (**Figure 4C**). Compared with CK group, ABA treatment promoted the expression of *TAGL1*, reaching 1.75 times that of the CK group at 7 d. Moreover, the expression of *TAGL1* responded rapidly to ABA, remaining substantially elevated levels compared with the CK group over the entire 48 h following ABA treatment (**Figure 4D**).

As shown in **Figure 4E**, a fluctuating trend of *MADS-RIN* expression levels was observed in CK group, with two peaks occurring at 4 d and 10 d. The expression level of *MADS-RIN* in ABA group showed an initial increase followed by a decrease, which regularly exceeded that of the CK group, reaching its maximum value at 7 d after ABA treatment, where it was markedly elevated (5.27-fold) compared with the CK group. Short-term effect results indicated that this gene responded relatively slowly to ABA, and its expression level increased suddenly at 4 h after ABA treatment but thereafter decreased compared with the CK group. It surpassed the CK group again at 24 h after treatment but was significantly lower than the CK group at 48 h (**Figure 4F**). The expression level of *CNR* in CK group showed an initial increase followed by a decrease (**Figure 4G**). In ABA group, however, it showed a fluctuating pattern. The expression level of *CNR* in ABA group was considerably elevated compared with the CK group at 1, 4, and 10 d after ABA treatment, but significantly lower than the CK group at 7 d. As shown in **Figure 4H**, *CNR* gene responded relatively slowly to ABA, and its expression was notably elevated compared with the CK group at 4 h after ABA treatment, dramatically diminished relative to the CK group at subsequent time periods, and substantially increased compared with the CK group at 24 h and 48 h.

The expression of *MADS1* showed an overall downward trend in two groups (**Figure 4I**). Furthermore, the expression in ABA group was inferior to that in the CK group, particularly at 1 d and 7 d, where it was 0.55- and 0.34-fold that of the CK group, respectively. Moreover, the gene responded rapidly to ABA regulation, remaining consistently lower than that in the CK group throughout the 48 h period after ABA treatment (**Figure 4J**).

The above results demonstrated that ABA promotes the expression of key transcription factor genes (*FUL1, TAGL1, MADS-RIN* and *CNR*), which positively regulate tomato ripening, while suppressing the expression of *MADS1*, a negative regulator of ripening.

### 3.6 Y1H assay

Based on the analysis results of the promoter sequence with PlantCARE, the selected gene sequence lengths and the precise ABRE positions are detailed in **Table 1**. Given that *ACS4* is an essential gene in ethylene biosynthesis pathway and there are two AREB elements exist in the different positions within the 2000 bp upstream sequences, its promoter was cut into two segments.

**Figure 5:**
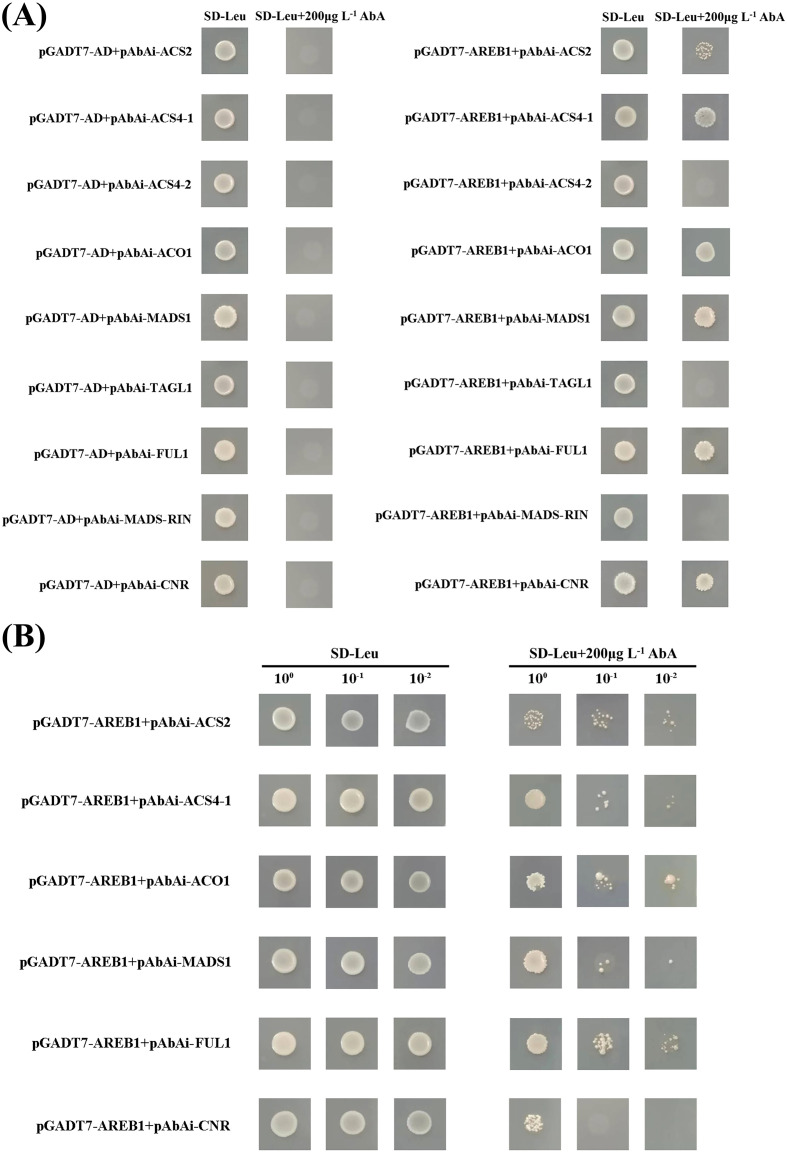
Y1H assay analysis of the interaction between SlAREB1 and ethylene biosynthesis genes. **(A)** Interaction verification of SlAREB1 and ethylene biosynthesis genes. **(B)** Validation of the positive clones by rotation experiment.

As shown in **Figure 5A**, the yeast co-transformed with the vector carrying SlAREB1 and the bait genes *ACS2*, *ACS4-1*, *ACO1*, *MADS1*, *FUL1*, and *CNR* grew on the SD/-Ura plates containing 200 μg L^-1^ AbA, which indicates that SlAREB1 may interact with the promoter of *ACS2*, *ACS4-1*, *ACO1*, *MADS1*, *FUL1*, and *CNR*. Conversely, the yeast co-expressing the SlAREB1 vector with the bait genes *ACS4-2*, *TAGL1*, and *MADS-RIN* failed to grow under the same selection conditions, suggesting no interaction between them.

To avoid false positive results, the yeast cultures were diluted to concentrations of 10^-1^ and 10^-2^ for the rotary verification, and similar results were obtained (**Figure 5B**). Therefore, it was preliminarily confirmed that SlAREB1 might interact with the promoters of *ACS2*, *ACS4-1*, *ACO1*, *MADS1* and *FUL1*.

### 3.7 Dual luciferase assay

**Figure 6:**
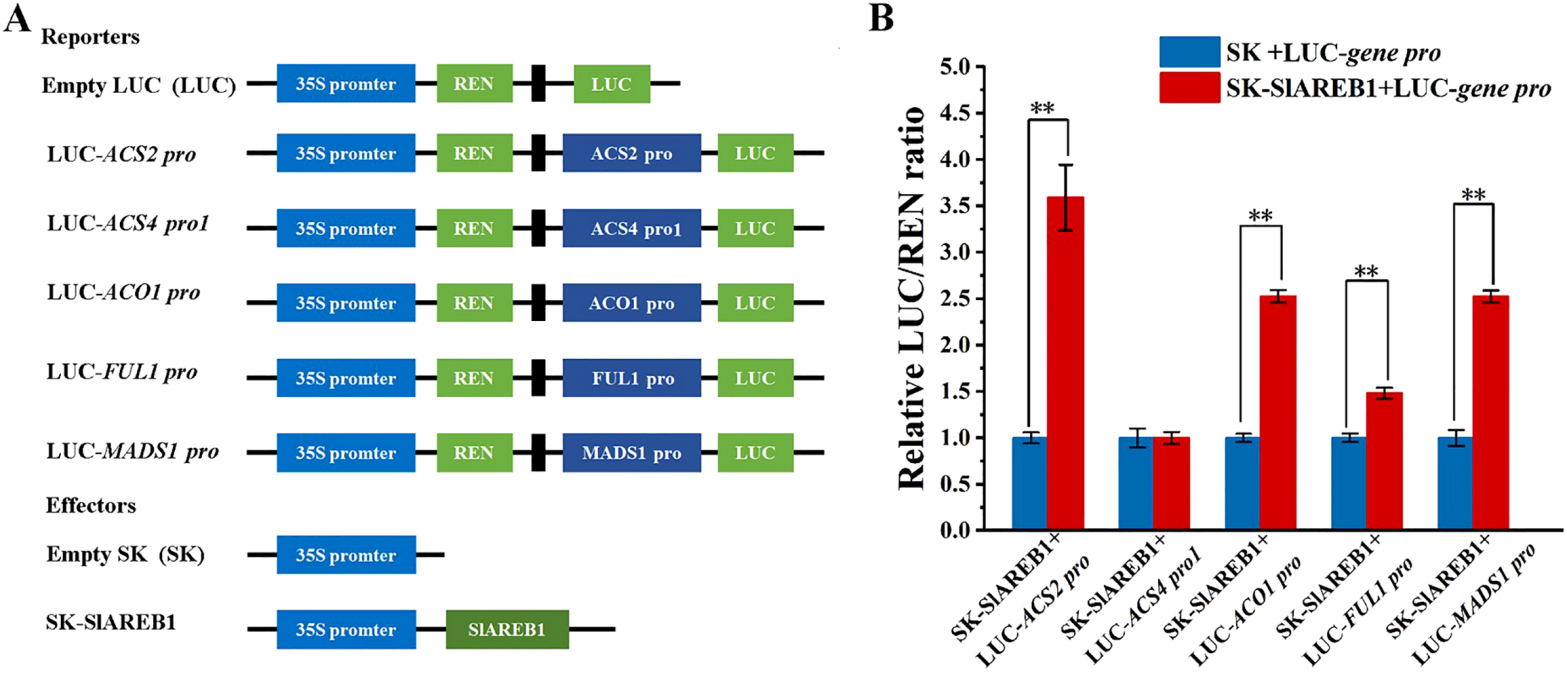
Dual luciferase analysis of the interaction between SlAREB1 and selected genes involved in tomato fruit ripening and the ethylene biosynthesis. **(A)** Constructions of effector and reporter utilized in the dual luciferase experiment. **(B)** SlAREB1 stimulates the promoter activity of *ACS2*, *ACS4-1*, *ACO1*, *FUL1*, and *MADS1* by dual luciferase experiment. SK+LUC refers to the double negative control, empty + promoter was a negative control, the error line represented the standard deviation of the mean value between the six parallels, and the asterisk (**) indicated a significant difference between no-load and SlAREB1 (*p* < 0.01).

To further validate the interaction between SlABEB1 and the selected genes involved in tomato fruit ripening and the ethylene biosynthesis, dual luciferase assays were performed according to the results of Y1H screening. As illustrated in **Figure 6A**, the effector vector SK-SlAREB1 and the reporter vectors LUC-*ACS2 pro*, LUC-*ACS4-1 pro*, LUC-*ACO1 pro*, LUC-*FUL1 pro*, and LUC-*MADS1 pro* were constructed according to the analysis results of the promoter sequence with PlantCARE. SlAREB1 exhibited transcriptional activation effects on *ACO1*, *ACS2*, *FUL1*, and *MADS1*, with LUC/REN ratios 2.5, 3.6, 1.5, and 2.5 times higher than those in CK group, respectively, while no interaction was observed with *ACS4-1* (**Figure 6B**).

**Figure 7:**
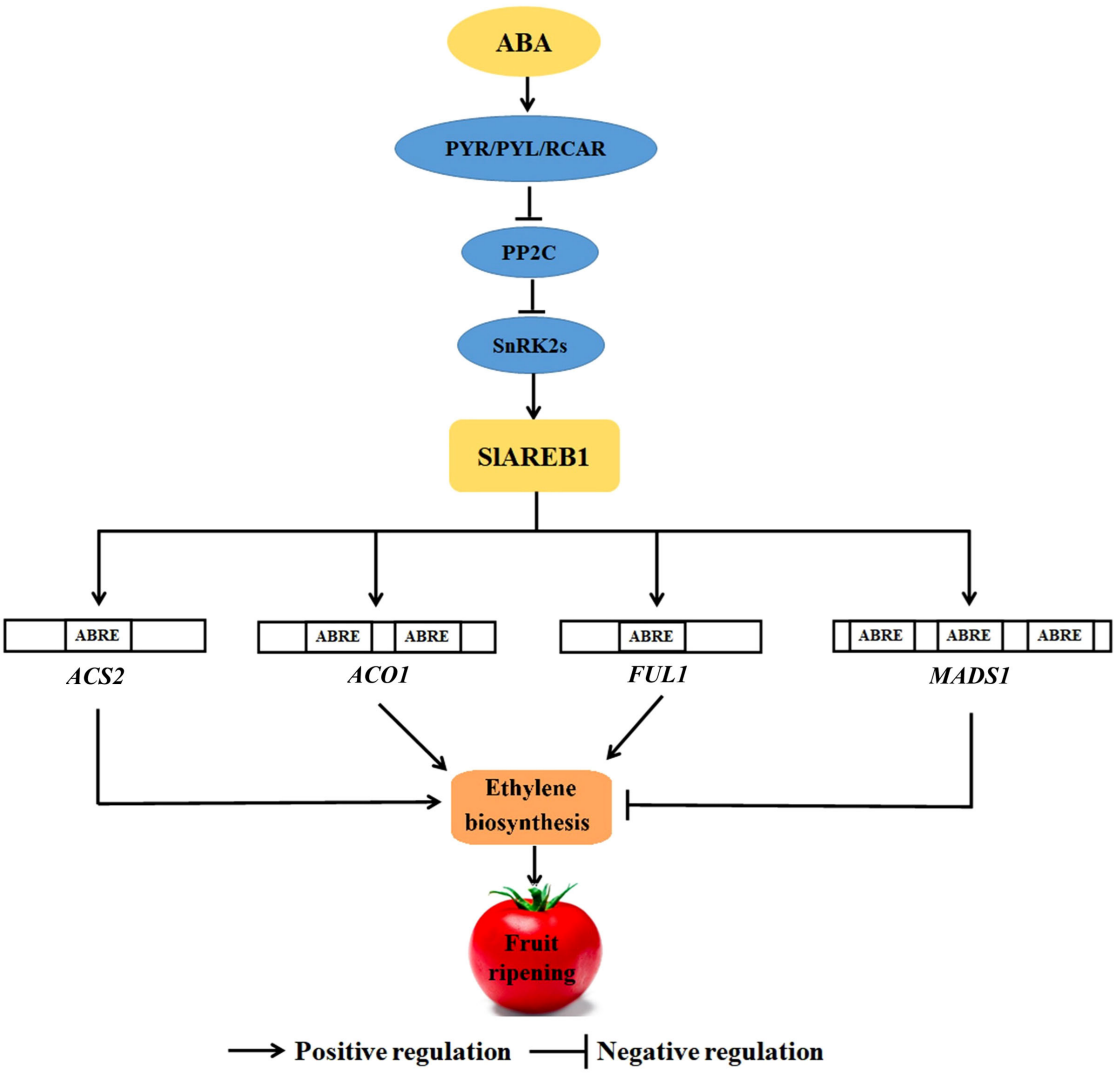
The molecular model of SlAREB1-mediated regulation of ABA on ethylene biosynthesis.

## 4 Discussion

Fruit ripening is a complex process genetically regulated by factors mediating ethylene biosynthesis, pigment accumulation, and cell wall metabolism (Kou et al., 2018). Color development, firmness loss, and ethylene production serve as critical indicators for evaluating tomato fruit ripening (Tao et al., 2022b). In this study, ABA treatment accelerated color transition and firmness loss, as well as increased ethylene production in tomato fruit, corroborating previous findings (Tao et al., 2022b; Wu et al., 2018a) and further validating ABA’s role in promoting tomato fruit ripening.

As a downstream responsive factor in the ABA signaling pathway, SlAREB1 mainly involves in regulating the expression of stress-related genes and the fruit ripening processes in tomato fruit (Bastias et al., 2011; Orellana et al., 2010). In this study, *SlAREB1* exhibited a peak expression at the mature green stage, and its expression was significantly higher than that in CK group from 7 d onward after ABA treatment. In addition, *SlAREB1* exhibited a rapid response to exogenous ABA treatment and the higher transcription abundance than that in CK fruit within 2 h after ABA treatment, suggesting that SlAREB1 has the potential to affect the ripening of tomato fruit and mediate the regulatory effects of ABA on the ripening process, which was consistent with the previous research results (Mou et al., 2018). Furthermore, studies revealed that *SlAREB1*-transgenic tomato fruit displayed significantly different expression patterns of genes involved in ethylene biosynthesis (Bastias et al., 2014). Specifically, compared with wild-type fruit, *SlAREB1*-transgenic tomato fruit had greater expression levels of *ACS2*, *ACO1*, and *ACO3*. Conversely, in the corresponding antisense inhibition plants, the expression of *ACO1* was lower than that in wild-type fruit. These results indicate that *SlAREB1* is modulated by ABA and plays a functional role in ABA-regulated ethylene biosynthesis.

Tomato fruit ripening is regulated by transcription factors and their downstream response genes, ethylene biosynthesis and signaling pathways (Wang et al., 2020). ACS and ACO are two key enzymes involved in ethylene biosynthesis (Chen et al., 2022; Isaacson et al., 2002). *ACS2*, *ACS4*, and *ACO1* are known as the predominant genes encoding key enzymes for ethylene biosynthesis (Kim et al., 2015; Kou et al., 2018). In this study, promoter analysis of these three genes revealed the presence of ABA-responsive elements AREBs, suggesting the potential regulatory roles of ABA on these genes. Further expression analysis demonstrated that the ABA treated group had significantly greater transcript levels for *ACS2*, *ACS4*, and *ACO1* genes compared with the CK group. Notably, the expression of *ACS2* was considerably elevated compared with the CK group as early as 12 h after ABA treatment, the expression of *ACS4* expression remained consistently higher in ABA treated group than that in CK group, and the expression level of *ACO1* was significantly higher than that in the CK group within one hour after ABA treatment. These observations indicate that exogenous ABA may stimulate ethylene biosynthesis by enhancing the expressions of *ACS2*, *ACS4*, and *ACO1* genes, aligning with previous findings (Mou et al., 2016). In *SlAREB1*-transgenic tomato fruit, the expression levels of *ACS2* and *ACO1* exceeded those in the CK group (Bastias et al., 2014). Also, in *SlAREB1* transient overexpression tomato fruit, the expression levels of *ACS2* and *ACO1* were consistently raised compared with the CK group, resulting in the increased ethylene production (Mou et al., 2018). The above results indicate that SlAREB1 may interact with *ACS2* and *ACO1* to promote ethylene biosynthesis. The expression peak of *SlAREB1* occurred earlier than that of *ACS2* and *ACO1*, and the promoter regions of *ACS2* and *ACO1* contain potential binding sites for SlAREB1, suggesting that SlAREB1 may act upstream of *ACS2* and *ACO1* and regulate their expression. This hypothesis was further verified via Y1H and double luciferase assays, which confirmed that SlAREB1 activates the transcription of *ACS2* and *ACO1*, that is, SlAREB1 promotes ethylene biosynthesis by interacting with *ACS2* and *ACO1*. In the results, the Y1H assay was positively interacted between SlAREB1 and *ACS4-1*, while the dual luciferase assay showed no significant difference. The phenomenon might be due to the requirement for certain cofactors present in the yeast system but absent in the tobacco system, or the strong basal activity of the promoter masking the activation effect of the transcription factor, or the occurrence of the transcriptional activation at an earlier or later time point. Although the positive regulation of ethylene biosynthesis by SlAREB1 through interaction with *ACS2* and *ACO1* has been well established, the experimental results concerning *ACS4* require further validation.

In tomato fruit, existing studies demonstrate that certain transcription factors affect fruit ripening by modulating ethylene biosynthesis. Positive regulators of tomato ripening include RIN (Li et al., 2011), CNR (Manning et al., 2006), TAGL1 (Itkin et al., 2009; Vrebalov et al., 2009), and FUL1 (Fujisawa et al., 2014; Wang et al., 2014a), which directly regulate key genes related to ethylene biosynthesis or indirectly modulate ethylene production by interacting with other critical transcription factors. Promoter analysis using PlantCARE in this study revealed ABA-responsive elements AREBs exist in the promoters of these genes, suggesting the potential regulation of ABA on them. The qRT-PCR results revealed that exogenous ABA treatment generally induced the expression levels of *FUL1*, *TAGL1*, *MADS-RIN* and *CNR*, compared with those in CK group. Notably, *FUL1* and *TAGL1* exhibited rapid responses to ABA, exhibit higher expression levels in ABA treated group than that in CK group within a short period of time, suggesting ABA treatment may affect ethylene biosynthesis by upregulating the expression of these genes, thereby modulating tomato fruit ripening. MADS1 negatively regulates fruit ripening by inhibiting ethylene biosynthesis through direct or indirect interaction with RIN (Dong et al., 2013). In this study, ABA treatment consistently inhibited the expression of *MADS1* throughout the storage period, with particularly significant suppression observed at 1 d and 7 d after ABA treatment. The gene responded rapidly to ABA, with its expression level was lower than that in CK group immediately after ABA treatment, indicating ABA may promote ethylene biosynthesis by repressing the expression of *MADS1*, thereby affecting tomato fruit ripening. While results of the Y1H and double luciferase experiments indicated that SlAREB1 interacts with the promoters of *FUL1* and *MADS1*, and activates the transcription of *FUL1* and *MADS1*, confirming that SlAREB1 positively regulates ethylene biosynthesis by interacting with them.

The existing studies indicate that *MADS1* is a negative regulator of ethylene biosynthesis, while SlAREB1 functions as a positive regulator. This apparent contradiction may stem from two potential explanations: (1) *MADS1* expression is not exclusively determined by SlAREB1. SlAREB1 may promote the expression of an inhibitory transcription factor that subsequently represses *MADS1* expression and finally promotes ethylene biosynthesis. (2) The activating effect is superior to the inhibitory effect. In this study, SlAREB1 was identified as an activator of three ethylene biosynthesis-related genes (*ACS2*, *ACS4*, and *ACO1*). Mou et al. (2018) demonstrated that SlAREB1 activated *NOR* to enhance ethylene biosynthesis as well, with ABA treatment enhancing the expression of *NOR* during tomato fruit ripening. The interaction between SlAREB1 and these genes may have a much greater promoting effect on ethylene biosynthesis than the inhibitory effect produced by the interaction with *MADS1*.

## 5 Conclusions

Based on the aforementioned findings, we constructed a molecular model of ABA’s modulatory role in SlAREB1-mediated ethylene biosynthesis (**Figure 7**). That is, SlAREB1 promotes ethylene biosynthesis through direct interaction with *ABRE cis*-acting elements located in the promoter regions of key ethylene biosynthesis genes (*ACS2* and *ACO1*) and the positive fruit ripening regulatory factor gene *FUL1*, and activating their transcription, thereby accelerating fruit ripening. Additionally, SlAREB1 binds to the ABRE element in the *MADS1* promoter region and activates its transcription, which inhibits ethylene biosynthesis. However, the ultimate regulatory effect remains the promotion of tomato fruit ripening. These findings establish a theoretical basis for comprehending the molecular mechanisms underlying hormonal interactions during fruit ripening.

## Data Availability

Data will be made available on request.
