# Supplementary material for: SlAREB1 regulates ethylene biosynthesis and mediates the effect of abscisic acid on postharvest ripening of tomato fruit
Source: Front Plant Sci. 2026 May 4;17:1773076. doi: 10.3389/fpls.2026.1773076 (PMC13180949; doi:10.3389/fpls.2026.1773076)
Supplement: Supplementary file 1 [file Table1.doc]

**Supplementary Table. 1.** Primer sequences used for quantitative real-time PCR analysis.

| Gene ID | Gene name | Primer | Sequence (5’-3’) |
| --- | --- | --- | --- |
| *SlAREB1* | Solyc04g078840 | -F | CGGGGAACTTTGGATTGCCG |
|  |  | -R | TGCCCATTAATCCCAGGCCC |
| *ACS2* | Solyc01g095080 | -F | TCGCGAGGATTCGGAGGTTC |
|  |  | -R | TGAGGGAGGAATAGGTGACG |
| *ACS4* | Solyc05g050010 | -F | CGTCTCCCCTGGATCTTCGT |
|  |  | -R | TCCTTGCAAGTGCGATCTCC |
| *ACO1* | Solyc07g049530 | -F | TTCTTGCGCCATCTTCCTACTT |
|  |  | -R | GCAAAATCTCTCATCACCTCTCTGT |
| *MADS1* | Solyc03g114840 | -F | TGCAGCACTTCAAGCATGGTG |
|  |  | -R | CCTTCGAGCTTAACGTGCCC |
| *TAGL1* | Solyc07g055920 | -F | TCTGCAAGCGTAGAAATGGGC |
|  |  | -R | TTGTCGTCGCAGTTTGGATGC |
| *FUL1* | Solyc06g069430 | -F | TACCTCCCCGGTAAGCTGGA |
|  |  | -R | AGCTGAATCAAGCTGGTGCTC |
| *MADS-RIN* | Solyc05g012020 | -F | ACATCATGGCATTGTGGTGAGC |
|  |  | -R | TGGTGCTGCATTTTCGGGTTG |
| *CNR* | Solyc02g077920 | -F | TCCTGGCCCACTTCTGTACG |
|  |  | -R | AGAACGTGTGAGGACCACCA |
| *Actin* | AK328563.1 | -F | TGTCCCTATTTACGAGGGTTATGC |
|  |  | -R | CAGTTAAATCACGACCAGCAAGAT |
